# Monthly Reports

**Published:** 1803-07-01

**Authors:** J. Ricards

**Affiliations:** No. 68, Hatton Street


### MONTHLY REPORTS.

*Cases admitted under the Care of the Surgeon of the Finsbury Dispensary, St. John's Square, Clerkenwell, from May* 10, *to June* 10, 1803.

| | | | |
|---|---|---|---|
| Ophthalmia - - - - | 1 | Spinæ Incurvatiæ - - | 3 |
| Abscessus Artuum - - | 4 | Hernia Incarcerata - - | 1 |
| ——— Psoas - - - | 1 | Dysecœa - - - - - - | 1 |
| Mastodynia - - - - - | 3 | Rachitis - - - - - - - | 1 |
| Ulcera Artuum - - - | 11 | Varix - - - - - - - | 1 |
| Vulnera - - - - - - | 4 | Tinea - - - - - - - | 1 |
| Contusiones - - - - | 9 | Psora - - - - - - - | 5 |
| Lues - - - - - - - - | 4 | | |
| Gonorrhœa - - - - | 2 | Total - | 53 |
| Scrophula - - - - - | 1 | | |

In a former Number of the Medical and Physical Journal, I related a case, and offered a few observations respecting the disease of Hydrocephalus; since that time several cases of the same nature have presented themselves to my knowledge, two of which were attended with or rather succeeded by circumstances of a rather peculiar aspect, but similar to each other, though in patients of a very different age. The first case was in a youth of seventeen, teen, who had every well-marked and authentic symptom of the disease Hydrocephalus, and which were extremely severe: they yielded however, after a short time, by the free use of drastic purgatives, vesication of the scalp, and slight mercurial friction. The peculiarity of the case did not occur till convalescence had far advanced; when, being considered as free from danger, he was suddenly afflicted with perfect dumbness, in which state he remained several weeks, apparently in good health, but unable to express his wants or his sensations, except by writing: he was in a state of extreme mental uneasiness from the consciousness of his situation, and was continually expressing a wish, by writing, that some person would cut his tongue, pointing at the same time to the frænum of the tongue, and endeavoured himself to obtain any sharp instrument for that purpose, more with a wish to satisfy his desire than with any expectation of advantage. A person made a slight wound with a pair of scissars in the frænum, when immemediately, to the astonishment of all present, he exclaimed with great joy, that he was well, and from that time he spoke as usual.

I shall make no remarks on this case, as the way in which such a circumstance could act is to me quite unintelligible.

The other case was in a child of three years old, who was afflicted with Hydrocephalus, which yielded to blisters on the head: Strong cathartics of elaterium, calomel, and gumboge, and the cinchona with iron towards the last. As he became convalescent, he likewise lost the power of utterance, which defect continued several weeks; but from which he recovered, not by any agency of an unaccountable operation, but by the rational application of electricity, which produced its good effects in a few repetitions.

In this latter case, the circumstance which I had remarked on a former occasion, viz. the necessity for very violent doses of cathartics in this disease, was peculiarly manifest; as the bowels of this child could not be acted upon by a less quantity for each dose than of elaterium, gr. ij; calomel and gumboge, aa. gr. v.

